# Direct-to-consumer Marketing to People with Hemophilia

**DOI:** 10.1371/journal.pmed.1001996

**Published:** 2016-06-14

**Authors:** Philip Kucab, Katelyn Dow Stepanyan, Adriane Fugh-Berman

**Affiliations:** 1 Department of Neurology, Detroit Medical Center, Detroit, Michigan, United States of America; 2 Department of Internal Medicine, University of California, Los Angeles, Los Angeles, California, United States of America; 3 Department of Pharmacology and Physiology, Georgetown University Medical Center, Washington, District of Columbia, United States of America

## Abstract

Adriane Fugh-Berman and colleagues describe how strategies similar to those used to market drugs to physicians are directed towards people with hemophilia.

**Summary Points**

- Factor manufacturers target many individuals with hemophilia using strategies similar to those used to market drugs to physicians, including the use of pharmaceutical sales representatives (“drug reps”), gifts, and financial opportunities.
- Additional strategies include sponsorship of camps for children, scholarships, and sponsorship of patient advocacy groups.
- The extent to which factor manufacturers affect discourse about treatments and payment decisions deserves public discussion.
- The question of whether industry targets individual patients with other diseases that require expensive, long-term treatments should be examined. Regulatory controls on industry interactions with patients should be considered.

Pharmaceutical companies routinely use gifts, friendship, and financial opportunities to befriend and obligate physicians and other prescribers in order to increase sales of targeted drugs [1–3]. Drugs are also promoted to patients through direct-to-consumer advertising and disease awareness campaigns [4–6]. Pharmaceutical promotion to people with hemophilia, however, goes far beyond other forms of promotion to consumers. This article documents the use of tactics that are usually reserved for prescribers instead being used to influence individual patients in the hemophilia community. Such tactics include gifts, financial opportunities, and one-on-one marketing through pharmaceutical representatives.

Worldwide, there are fewer than 500,000 people with hemophilia (PWH), including about 20,000 people in the United States [7,8]. Hemophilia is a rare X-linked genetic disease resulting in deficiencies in clotting factor VIII (hemophilia A) or IX (hemophilia B). While minor injuries often heal normally, major bleeding may follow surgery or trauma. Patients with severe hemophilia (<1% normal levels of factor) can bleed spontaneously with no known trauma. While the life expectancy of PWH has increased dramatically over the last 50 years, untreated bleeding episodes can result in severe joint damage, neurological damage, and premature death.

Factor concentrates, commercially available since the 1960s, have been self-administered by patients since the 1980s. Individual use of clotting factor varies widely. In any given year in the US, about a quarter (23%) of individuals with hemophilia use no clotting factor at all [9]. Adult patients with severe hemophilia A treat prophylactically (scheduled factor treatments regardless of bleeding) or episodically (factor treatments in response to bleeding episodes). In both cases, additional factor may be required after surgery or trauma.

## Costs and Uncertainties

Optimal strategies for several aspects of hemophilia treatment remain uncertain [10–13]. Current uncertainties include optimal dosages of factor and whether different therapies or regimens are more or less likely to induce alloantibodies (“inhibitors”).

Hemophilia is an expensive disease. Although the number of PWH is small, the hemophilia market is large: in 2011, according to the investment research firm Morningstar, the international market for hemophilia products was US$8.5 billion; products for patients with hemophilia A accounts for US$5.2 billion of that number [14]. In comparison, global sales of Lipitor (atorvastatin), the best-selling drug in the world, were US$12.5 billion in 2011 [15].

An actuarial study of 2008–2011 health care claims found that severe hemophilia A patients incur annual health care costs of about US$160,000 [16]; an analysis of employer-based insurance found similar results [14]. Direct costs vary with disease severity. A multicenter study of 222 patients from six federally supported hemophilia treatment centers (HTCs) found that average annual costs were US$59,101 for mild hemophilia and US$84,363 for moderate hemophilia. For severe hemophilia, average annual costs were US$201,471 for episodic treatment and US$301,392 for prophylactic treatment [17]. Clotting factor contributed 54% of total costs for patients with mild hemophilia and, at the other end of the spectrum, 94% for patients with severe hemophilia who were receiving prophylaxis [17].

In the US, Medicaid, Medicare, state programs, and private insurance usually cover medications, although patients may be responsible for copayments or deductibles. The government is the biggest payer: Medicare and Medicaid cover 30%–40% of male hemophiliacs and pay for about two-thirds of hospitalizations associated with hemophilia [14].

Six companies produce roughly 20 factor products. Some products are derived from human plasma; others are recombinant. No difference in clinical outcomes has been demonstrated among similar factor products [18,19]. Nonetheless, a 2002 analysis of prescriptions for hemophilia products in a sample of over 40 hospitals in the US found that escalating costs were attributable to a shift to newer, more expensive recombinant factor products [19]. Between 1992 and 2000, new products within an already existing category of factor were priced 68% higher than the previously marketed product [19].

Prophylactic treatment, in which patients are given regular doses of factor two or three times weekly to prevent bleeding, is now considered the standard of care. Compared with episodic or “on-demand” treatment, in which factor is administered after a bleed has started, prophylaxis increases the amount of factor used and subsequent costs [20–22]. About 65% of factor VIII consumption globally and 75% of sales in developed markets are for prophylaxis [14].

Inhibitors also increase treatment costs. Twenty-five to thirty percent of patients with severe hemophilia A and 3%–5% of people with hemophilia B develop inhibitory alloantibodies (“inhibitors”), which reduce treatment efficacy and increase morbidity and mortality [23]. It is unclear whether some types of products are less likely to induce inhibitors. Patients who develop inhibitors may undergo “immune tolerance induction,” an expensive treatment involving very large amounts of factor infused almost continuously over months or years [9].

A pharmacoeconomics study found that the annual median aggregate health care costs for patients with hemophilia A with inhibitors were US$325,780, more than triple the US$98,334 spent on those without inhibitors [21]. Among Medicaid-covered male patients with hemophilia A in 2008, the average annual total costs were US$142,987. For both Medicaid-covered individuals and those covered by employee-sponsored insurance, the presence of inhibitors tripled costs in adults; in children, the presence of inhibitors increased costs 6-fold [24]. Prophylaxis costs are also higher in those with inhibitors [25].

## Promoting Factor: Drug Reps, Gifts, Grants, and Jobs

PWH often make their own decisions about what product they use, which is acknowledged by factor manufacturers. A senior director of marketing at Baxter, quoted in a hemophilia newsletter, said, “From our research, about 50% of patients are involved in their own decision making. In other therapeutic areas, it’s only 5% to 20% of the time [26].”

A senior director at Bayer HealthCare stated, *“*Hemophilia patients tend to be highly organized and well connected, well read, and more savvy than the typical consumer…. We believe consumers are looking for a long-term relationship that extends well beyond the product or service itself” [26].

Industry begins establishing lifetime relationships with PWH during childhood. Baxter, for example, sponsors Camp Superfly, a summer camp for children with hemophilia [27]. Baxter sales representatives help staff some camps, providing an opportunity to establish personal relationships with young campers. A drug rep for Alpha Pharmaceuticals, another factor manufacturer, described working in a hemophilia camp: “There is a tight-knit community in the hemophiliac industry…. I like working with these people over the years, and building long-lasting rapport” [28].

In one of the authors’ (PK) personal experience, sales representatives from each company are assigned to individual patients. (PK has hemophilia; he is on the board of directors of the World Federation of Hemophilia USA, a consultant to the National Hemophilia Foundation, and an advisor to Bayer HealthCare.)

With other diseases, physicians are commonly recruited by industry as “key opinion leaders” to influence the prescribing of peers [1]. In hemophilia, however, patients and their families are recruited by factor manufacturers for employment, consulting roles, or advisory boards. A recent New York Times article notes that both patients and their relatives are hired by hemophilia drug manufacturers and the specialty pharmacies that dispense factor. The article quotes a representative from the National Hemophilia Foundation saying “There are a lot more patients that work in industry now than ever before.” [29]

Factor manufacturers also recruit people who are well connected in the hemophilia community, including staff and volunteers from patient advocacy groups and nurses from HTCs. Biogen Idec, which entered the hemophilia market in 2014, hired a Community Relations (“CoRe”) team that comprises “parents, advocates, friends, nurses, social workers, and people with hemophilia… Our goal is to be your partner, whether that means helping at chapter events, creating meaningful programs, or offering our experience to the community at large” [30]. Novo Nordisk convened a Consumer Council, a group of patients with inhibitors, their parents, and their caregivers who “are dedicated to serving as ambassadors for the inhibitor community.” The Consumer Council meets regularly “to discuss the needs of the inhibitor community and ways in which they can offer support through communication and education” [31].

Young adults with hemophilia are offered paid internships, college scholarships, awards, career counseling, and insurance counseling, recruited to consumer and professional advisory boards, and offered paid consulting opportunities. Branded toys designed for children and other promotional gifts are common at national hemophilia meetings.

Several companies offer grants for education. Novo Nordisk’s SevenSECURE program offers grants of up to US$500 per child annually “to pay for tutors, home schooling, or other things like swimming lessons, music classes, and sports” during grades K–12 (age 5–18) [32]. High school seniors, college students, and vocational students are eligible to compete for “Professor Ulla Hedner Scholarships,” which provide up to US$7,000 towards tuition or school expenses. “Adult Education Grants” of up to US$2,500 are available for adults “with congenital hemophilia with inhibitors, congenital factor VII deficiency, or acquired hemophilia” or “a primary caregiver” to “take courses,” “continue training,” or “learn skills for a new career” [32].

Pfizer’s Hemophilia Village website offers Soozie Courter Scholarships: annually, ten US$2,500 undergraduate scholarships and five US$4,000 graduate school scholarships to students with hemophilia. Scholarship recipients are selected by “a program selection committee of hemophilia healthcare providers.” The address for submitting applications is a public relations firm [33].

The New York Times article describes a case in which a pharmacy sales representative attempted to take a teenager with hemophilia out to dinner without a parent present [29]. In PK’s experience, adults with hemophilia are not only taken out to meals but paid to participate in focus groups and interviews. Industry representatives also establish relationships with parents and caregivers. In PK’s experience, parents of a child with hemophilia may be paid to counsel families with a newly diagnosed child.

Companies may provide copay programs, discounts, or assistance with persuading payers to cover specific therapies. Pfizer offers a month’s worth of free factor and a US$12,000 savings card that can be used for copays, deductibles, and coinsurance costs [34]. Pfizer’s RxPathways program provides “reimbursement support services” for its brand of factor. The site states that “If a claim is underpaid or denied, a Pfizer RxPathways Program representative can look into it on your behalf and explain the appeals process to you” [35]. Novo Nordisk’s SevenSECURE program offers assistance with payment of insurance premiums, medical expenses, and dental expenses, as well as help with appealing “denials of coverage to your insurance company” to patients with inhibitors, factor VII deficiency, or acquired hemophilia [32]. Similarly, Baxalta (a new company spun off from Baxter International in 2015) offers the Coverage, Assistance, Resources, and Education (CARE) program, which states that “you'll have people on your side to help you achieve coverage for treatment”; the program also provides “Co Pay or Coinsurance… support” that “may be available to reduce your out-of-pocket costs associated with your Baxalta treatment” [36].

Personal, health, and career guidance are also offered. For example, Baxter hosts a CEO (career, education, and opportunities) event that provides a full day of career guidance for 15–20-year-olds [27]. Baxalta’s NAVA program provides “highly personalized information and tools to provide education and support that are specific to your needs,” including “A NAVA Partner who'll help you set—and achieve—goals that can help you pursue your life interests” [37]. Patients or family members are assigned “A NAVA Mentor…someone who can lend an ear, based on their own real-life experiences” [38]. Bayer offers “Living Fit! A Joint Effort,” a program that “helps young people with hemophilia A get active and live fit” [39]. The Bayer Virtual Walk for Hemophilia, an interactive online program, offers up to US$60,000 in sponsorship funds for “virtual walkers” [40].

Several companies also offer electronic factor logs. Bayer, which sponsors the Living with Hemophilia website [39], offers FactorTrack, a mobile application that helps patients to track and record infusions. Additionally, the app provides reminders for a prophylactic regimen. Pfizer offers HemMobile, an app that “lets you manage infusions and bleeds from the palm of your hand” and “Send reports to your doctor” [41].

Baxter once owned Advoy, an “electronic, web-based patient bleeding and infusion diary” that “supports therapy management with secure two-way messaging between patients and their HTC.” Baxter recently donated Advoy to the American Thrombosis and Hemostasis Network (ATHN), which is a nonprofit organization supported by industry. Baxter has stated the rationale for the donation is that “Some have expressed the desire to support an information infrastructure that is not owned by industry” [42]. Novo Nordisk and Baxter Bioscience are credited as funders in ATHN’s 2010 annual report, the only annual report on the website as of January 2015 [43].

## Sponsored Organizations, Meetings, Events, and Publications

Baxter sponsors many activities through hemophilia nonprofits throughout the US [27]. For example, Baxter Bioscience funds the Hemophilia Association of New Jersey to organize advocacy workshops and administer grants to “support state advocacy initiatives that are geared toward maintaining and/or expanding access to high quality care for persons with hemophilia.” Since 2005, 84 grants totaling about US$1,975,000 have been given out. Some funded projects sought to “Protect access to medicine and services through state legislation” and “Conduct grassroots advocacy campaigns, directed at key decision makers, in an effort to preserve quality of care” [44]. Baxalta donated at least US$40,000 to the New England Hemophilia Association for fiscal year 2015 [45].

Since 2005, Novo Nordisk has funded “Inhibitor Education Summits,” which educate patients with inhibitors on medical, psychosocial, insurance, and access-to-care issues [46,47]. The conference is currently funded by Novo Nordisk and Baxalta through an unrestricted grant to the National Hemophilia Foundation [48]. Novo Nordisk and Baxalta also fund regional Inhibitors meetings [49]; Novo Nordisk also funds Inhibitor webinars based on the Education Summits [50]. Pfizer has disclosed payments to hemophilia organizations in at least 26 states [51–53].

The National Hemophilia Foundation (NHF), a nonprofit national advocacy organization that dates back to 1948, advocates on behalf of people with bleeding disorders. NHF supports basic science and clinical fellowships and disseminates medical information and recommendations relevant to the bleeding disorders community. In 2013, the NHF reported revenues of US$15.3 million; US$14.1 million came from grants and contributions [54]. Information is not available from all companies, but Pfizer disclosed contributing a total of US$1,076,500 to NHF in 2013 [51], US$320,000 in 2014 [52], and US$1,115,000 in 2015 [53].

Advertising provided more than a million dollars in revenue as well [54]. A study of advertisements in *HemAware*, the NHF’s bimonthly magazine, concluded that more information was presented about benefits than harms. On average, type size regarding benefits was several millimeters larger than that devoted to risks [55]. A majority of raters found that about two-thirds of the advertising claims were based on low-quality evidence; about one-third were rated as not evidence based.

The effect of industry funding on what advocacy groups say has been raised previously [56–59]. A United Kingdom charity, the Multiple Sclerosis (MS) Trust, celebrated a decision by the National Health Service in Wales to cover nabiximols, a cannabinoid spray that treats MS-associated spasticity, without disclosing funding from Bayer, which markets the drug in the UK [56]. The Canadian Arthritis Association allowed its logo to be used on newspaper supplements claiming safety advantages for Vioxx (rofecoxib) and Celebrex (celecoxib) without disclosing funding from coxib manufacturers; fact sheets on the Association’s website failed to mention cardiovascular risks of coxibs [59].

The importance of patient groups to factor manufacturers was made clear at a Pfizer-sponsored satellite symposium (an event before, during, or after, but not officially part of, the main meeting) at the 2013 European Haemophilia Consortium Congress in Bucharest, Romania, called “Changing the policy landscape: haemophilia patient involvement in healthcare decision-making.” A summary written by a medical education company paid by Pfizer notes that “Differences in the supply and use of replacement factor concentrates, access to comprehensive care and the option of prophylaxis are vast and unacceptable. Collaboration and cooperation between patient organisations, clinicians, payers and industry is key to advocating for optimal care for people with haemophilia.” [60]

The summary, in an industry-funded journal supplement (a non-peer-reviewed bonus publication that bears a journal’s name) [2], also states, “Small differences in measurable outcomes can have a significant impact on patient quality of life, and although clinical outcomes are the cornerstone of assessing efficacy of care, the emphasis must also be placed on real-world outcomes that are meaningful to patients” [60].

Such statements raise the question of whether patient groups are being influenced to advocate for specific treatments or regimens and whether patients’ personal perspectives are being used to influence payers to cover therapies for which there is no scientific evidence of superiority.

## Discussion

To our knowledge, this is the first publication that documents the targeting of individual patients with marketing tactics that mirror and extend the strategies typically used to influence physicians. Similarly to clinicians, PWH are targeted via sales representatives, grants, gifts, meals, services, and consulting opportunities. Additional strategies include camps, support groups, scholarships, and insurance assistance programs. Factor manufacturers and distributors have fostered dependency of patients with hemophilia by providing jobs, scholarships, psychological support, and organizational support.

Consumer advocacy groups can help pharmaceutical companies by building demand for new treatments, facilitating regulatory approval of a therapy, and providing quotes for corporate press releases, as well as through many other services [61]. Companies may also recruit patient advocacy groups to convey industry-friendly messages to other patients, to payers, or to health care agencies.

While consumer voices are indeed important, the close relationship between patient advocacy groups and industry may distort public and medical discourse. Industry may use industry-supported advocacy groups to pressure payers and legislators to cover more expensive formulations or dosing regimens. Whether more expensive products or formulations are worth the additional expense—or simply increase profits for manufacturers with little additional benefit—is subject to debate and requires further scrutiny. Even when a group does not advocate an industry-friendly viewpoint, public discourse may be affected. As noted by Sharon Batt, an expert on relationships between industry and advocacy groups, “pharma funding buys silence on matters that groups speaking for patients ought to bring to the table” [58].

High factor prices are a significant expense for both public and private payer systems and affect the financial stability of patients who pay for part or all of their services out of pocket. The factor industry has been called an “oligopoly” [20] and “fundamentally non-competitive” because several companies hold the majority of market share [19]. Larger vial sizes, increased ease of reconstitution, or longer-acting products may increase treatment adherence. In most cases, however, the advantage of more expensive products or formulations is questionable. Industry expends massive promotional resources on creating and maintaining patient loyalty to specific brands. The strategy is effective: the rate at which patients switch from one therapy is less than 10% [14].

Direct and indirect promotion aimed at increasing individual factor use in a fixed patient pool may compromise objective assessment of treatment, prophylaxis, and dosing regimens. The impact of relationships between factor manufacturers and patients—as well as their parents and caregivers—currently flies under the radar of regulatory scrutiny.

## Recommendations

Robust scientific debate regarding best treatments is healthy, but the extent to which factor manufacturers affect discourse about treatments and prophylaxis among researchers and PWH deserves public discussion. The extent to which manufacturers use patient advocacy organizations as mouthpieces for marketing messages must also be addressed. Additionally, the effect of industry-generated friendship, community, scholarships, job offers, organizational support, and other gifts on patient perceptions of what are the best therapies should be assessed.

There are many possible reasons for lack of clarity on treatment and prophylaxis regimens, including lack of funding and the intrinsic difficulties in conducting adequately powered trials with a limited patient pool. The contribution of sponsored research to this confusion should be explored. Best practices should be established by entities that are not funded by industry.

Ideally, hemophilia physicians and researchers would eschew speaking and consulting arrangements with factor manufacturers and distributors. Patients should make health care decisions in partnership with health care providers who have no conflicts of interest. Patients and health care providers should rely on non-industry-funded sources of information, and factor manufacturers should have no role in funding patient groups, medical groups, or continuing medical education. This would require hemophilia organizations to forego industry funding, which may be difficult for a community that has become accustomed to industry support of both organizations and individuals. It is patients and payers, however, who may suffer economic harms from industry promotion.

Federal funding of both hemophilia research and comprehensive care should be increased. Government funding is vital for comparative effectiveness research, as objective assessment of treatments and prophylaxis regimens is unlikely to come from companies competing for a limited market.

The question of whether industry targets individual patients with other diseases that require expensive, long-term treatments remains to be examined. Regulatory controls on industry interactions with patients should be considered.

## Abbreviations

ATHN: the American Thrombosis and Hemostasis Network
CARE: Coverage, Assistance, Resources, and Education
CEO: chief executive officer
CoRe: Community Relations
MS: multiple sclerosis
NHF: National Hemophilia Foundation
PWH: people with hemophilia
HTC: hemophilia treatment center
